# Biologic Monitoring to Characterize Organophosphorus Pesticide Exposure among Children and Workers: An Analysis of Recent Studies in Washington State

**DOI:** 10.1289/ehp.8022

**Published:** 2005-07-06

**Authors:** Richard A. Fenske, Chensheng Lu, Cynthia L. Curl, Jeffry H. Shirai, John C. Kissel

**Affiliations:** 1 Department of Environmental and Occupational Health Sciences, School of Public Health and Community Medicine, University of Washington, Seattle, Washington, USA; 2 Department of Environmental and Occupational Health, Rollins School of Public Health, Emory University, Atlanta, Georgia, USA; 3 Integral Consulting Inc., Boulder, Colorado, USA

**Keywords:** agricultural communities, agricultural workers, biologic monitoring, children, dialkylphosphates, organophosphorus pesticides, pesticide exposure

## Abstract

We examined findings from five organophosphorus pesticide biomonitoring studies conducted in Washington State between 1994 and 1999. We compared urinary dimethylthiophosphate (DMTP) concentrations for all study groups and composite dimethyl alkylphosphate (DMAP) concentrations for selected groups. Children of pesticide applicators had substantially higher metabolite levels than did Seattle children and farmworker children (median DMTP, 25 μg/L; *p* < 0.0001). Metabolite levels of children living in agricultural communities were elevated during periods of crop spraying. Median DMTP concentrations for Seattle children and farmworker children did not differ significantly (6.1 and 5.8 μg/L DMTP, respectively; *p* = 0.73); however, the DMAP concentrations were higher for Seattle children than for farmworker children (117 and 87 nmol/L DMAP, respectively; *p* = 0.007). DMTP concentrations of U.S. children 6–11 years of age (1999–2000 National Health and Nutrition Examination Survey population) were higher than those of Seattle children and farmworker children at the 75th, 90th, and 95th percentiles. DMTP concentrations for workers actively engaged in apple thinning were 50 times higher than DMTP concentrations for farmworkers sampled outside of peak exposure periods. We conclude that workers who have direct contact with pesticides should continue to be the focus of public health interventions and that elevated child exposures in agricultural communities may occur during active crop-spraying periods and from living with a pesticide applicator. Timing of sample collection is critical for the proper interpretation of pesticide biomarkers excreted relatively soon after exposure. We surmise that differences in dietary exposure can explain the similar exposures observed among farmworker children, children living in the Seattle metropolitan area, and children sampled nationally.

Children may experience greater risks from pesticide exposures than adults because of behavioral, dietary, and physiologic characteristics associated with development (National Research Council 1993). University of Washington researchers began an investigation of children’s exposure to pesticides in 1991, with particular emphasis on organophosphorus (OP) pesticide exposures of presumed high-risk populations, such as children of pesticide applicators, children of farmworkers, and children living in agricultural regions with substantial agricultural pesticide use (Curl et al. 2002; Fenske et al. 2002; Koch et al. 2002; Loewenherz et al. 1997; Lu et al. 2000, 2001; Simcox et al. 1999). These studies have suggested that children with parents who apply pesticides in agriculture and who live in agricultural areas during active crop spraying receive higher OP pesticide exposure than do other children.

Each of these studies has employed biologic monitoring of urinary dialkylphosphate (DAP) metabolites to yield information on OP pesticide exposure. Biologic monitoring is a valuable tool in exposure assessment, allowing for integrated measurement of exposure from all pathways and routes. Biologic monitoring has been used effectively to evaluate exposures to populations across studies and across time (Barr et al. 2004a; National Research Council 1991). This approach has been used in many regional studies to determine OP pesticide exposures among young children (Adgate et al. 2001; Aprea et al. 2000; Heudorf and Angerer 2001; Heudorf et al. 2004; Mills and Zahm 2001; Morgan et al. 2005; Royster et al. 2002; Shalat et al. 2003).

More than 30 OP pesticides are registered for use in Washington State, and many of these pesticides do not have urinary metabolites that can be considered selective. When we began the Washington State biologic monitoring studies in 1994, selective metabolites were not available for azinphosmethyl and phosmet, the primary OP pesticides used in the region. We therefore developed an assay for the DAP metabolites (Moate et al. 1999). The DAP metabolites are the common products of OP pesticide metabolism and integrate exposure from most registered OP pesticides (Barr et al. 2004a). The National Center for Environmental Health now includes the DAPs among the metabolites assayed as part of the National Health and Nutrition Examination Survey (NHANES). Their most recent reports present DAP levels for participants 6–59 years of age and thus provides information on current levels in the general U.S. population [Centers for Disease Control and Prevention (CDC) 2003, 2005]. One concern in the use of exposure biomarkers is the possibility that the compounds being assayed could appear in food or the environment as degradation products (Lu et al. 2005; Morgan et al. 2005). As with any breakdown products that are urinary biomarkers of toxicant exposure, if exposure to the breakdown product occurs, and if this compound is absorbed efficiently into the body and excreted in the urine unchanged, then its appearance in urine samples could confound interpretation of such measurements. To date, however, no published studies have demonstrated that DAPs behave in this fashion.

The purpose of the present analysis was to examine OP pesticide metabolite concentrations in five Washington State studies, conducting both qualitative and quantitative (statistical) comparisons, with special attention to issues of study design and sampling that make such comparisons problematic. The five studies include apple thinners exposed to OP pesticides when reentering fields after applications (Simcox et al. 1999); children of agricultural pesticide applicators, many of whom lived near pesticide-treated farmland (Loewenherz et al. 1997; Lu et al. 2000); children living in the Seattle metropolitan area whose parents were not occupationally exposed to pesticides (Lu et al. 2001); children living in an agricultural community whose parents were not involved significantly in agricultural production (Koch et al. 2002); and children living in households with adults employed as farmworkers in a variety of agricultural activities (Curl et al. 2002; Thompson et al. 2003). The aforementioned biologic monitoring data from the CDC provide an opportunity to draw comparisons between the study populations and the general U.S. population (Barr et al. 2004a; CDC 2003). It is our hope that this analysis will prove useful in the development of future study designs and sampling plans for population-based pesticide exposure studies.

## Materials and Methods

We used seven populations enrolled in five independent studies, all of which took place in Washington State between 1994 and 1999, in this analysis. Our previous reports included detailed descriptions of population recruitment, sample collection, and sample analysis (Curl et al. 2002; Koch et al. 2002; Loewenherz et al. 1997; Lu et al. 2000, 2001; Simcox et al. 1999; Thompson et al. 2003). Tables 1 and 2 provide geographic location, number and age of participants, and number of samples collected in these studies and describe relevant occupational or para-occupational factors, as well as relationships between sampling time and active crop spraying with OP pesticides.

### Study designs.

**Figure 1:**
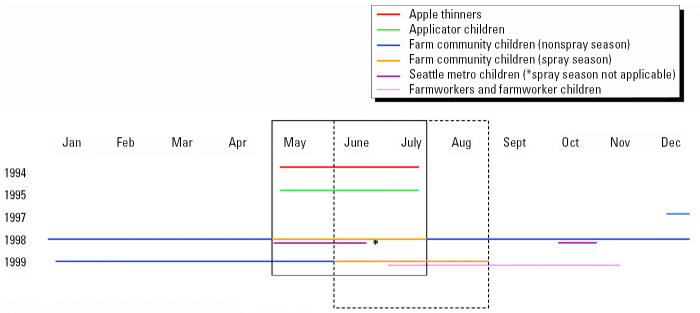
Timeline for urine sampling. The solid box indicates the agricultural dimethyl OP pesticide spray season for 1994, 1995, and 1998. The dashed box indicates the agricultural dimethyl OP pesticide spray season for 1999.

Three studies included in this analysis were cross-sectional in design. The first was conducted in Douglas and Chelan Counties in the Wenatchee Valley region of central Washington State. The primary industry in this region is tree fruit production, and the area includes many small family orchards. Forty-nine families of pesticide applicators participated in this study, including 72 children between the ages of 2 and 6 years (applicator children). Each child provided two individual urinary voids separated by 3–7 days. Sample collection occurred between May and July of 1995, during the time that dimethyl OP pesticides were applied to control the codling moth (Figure 1). A complete discussion of the methods for this study is provided by Loewenherz et al. (1997) and Lu et al. (2000).

The second cross-sectional study occurred in the Seattle metropolitan area (King and Snohomish counties). Participants included 110 children 2–5 years of age from 96 families. Parents of children enrolled in this study were not occupationally exposed to pesticides. Participating families were originally separated into one of two groups based on socioeconomic status, but no difference was observed in OP pesticide metabolite levels based on community or family income (Lu et al. 2001). These children were considered a single group (Seattle children) in the present analysis. Each child provided one sample in the spring of 1998 (May–June) and another in the fall of 1998 (September–November), as indicated in Figure 1. Methods for this study are described by Lu et al. (2001).

The third cross-sectional study was designed as a baseline survey of communities enrolled in a multiyear community intervention project. It included 24 communities in the Yakima Valley region of Washington State. This area, like the Wenatchee Valley, is a very productive agricultural region that includes tree fruit production. Dimethyl OP pesticides are applied in the late spring to protect against the codling moth, and other OP pesticides are used as a part of agricultural production. Two hundred eighteen households including both an adult farmworker and a child 2–6 years of age were enrolled in this study. The farmworkers were engaged in a variety of work tasks, such as harvesting, pruning, planting, weeding, and irrigating. About 20% reported some involvement in pesticide mixing, loading, or application. Urine samples were provided by 213 adults (farmworkers) involved in agricultural tasks such as harvesting, weeding, and pesticide applications, as well as by 211 farmworker children. Urine samples for farmworkers and farmworker children consisted of a composite of either two or three voids, each separated by a minimum of 3 days, and all collected within a 2-week period. These samples were collected between July and October of 1999 (Figure 1). Methods for this study are described by Curl et al. (2002) and Thompson et al. (2003).

The two remaining studies included in this analysis focused on relatively small populations but sampled participants repeatedly over time. Both were conducted in the Wenatchee region of Washington State. The first study investigated dimethyl OP pesticide exposures in a group of 20 adult apple thinners; sampling took place between May and July of 1994. These workers entered treated fields soon after OP pesticide applications (Figure 1). Each worker provided between 7 and 21 individual voids; a total of 293 voids from these workers were included in this analysis. Methods for this study are described by Simcox et al. (1999).

The last study included in the analysis was a longitudinal study of OP pesticide exposures among preschool children living in an agricultural community. A group of 44 children 2–5 years of age provided biweekly urine samples for up to 1 year. Sampling for this study was conducted between December 1997 and August 1999. This encompassed two periods of active crop spraying (Figure 1). Koch et al. (2002) reported that dimethyl OP pesticide metabolite levels were significantly elevated in these children during the periods of active crop spraying. Therefore, samples collected during periods of active crop spraying (farm community children, spray season) were considered separately from those collected during other times of the year (farm community children, nonspray season). This analysis included 274 samples collected from the 44 children during the spray season and 694 samples collected during the nonspray season. Methods for this study are described by Koch et al. (2002).

### Sample analysis.

Urine samples collected in all studies were analyzed for DAP metabolites common to most OP pesticides. We selected dimethyl DAP metabolites as the focus for comparisons across studies because they were substantially and consistently higher than diethyl metabolites in all studies. Dimethylthiophosphate (DMTP) was selected as the best comparative indicator of exposure across all studies. DMTP has been found to be the dominant of the three dimethyl DAP metabolites in samples analyzed in all of our studies (Curl et al. 2002, 2003; Koch et al. 2002; Lu et al. 2000, 2001; Simcox et al. 1999), as well as in the 1999–2000 NHANES population (NHANES III; CDC 2003).

Sample analysis for all of the studies was conducted by the University of Washington Environmental Health Laboratory following the procedure described by Moate et al. (1999). This procedure involved solid-phase extraction, azeotropic distillation, and derivatization with pentafluoro(methyl)benzylbromide, followed by gas chromatographic detection. That all samples were analyzed within the same laboratory provides reassurance that urinary metabolite levels can be compared directly, because results of such assays have been shown to vary across laboratories (James et al. 2003).

Samples with metabolite concentrations below the limit of detection (LOD) were assigned one-half the value of the LOD for this analysis. The LOD in the later studies was lower than for the 1994 study of apple thinners and the 1995 study of applicator families (e.g., 40 μg/L LOD for DMTP in 1994, 20 μg/L in 1995, 1.1 μg/L in 1998 and 1999). To reduce the relative impact of this higher detection limit, we assumed the lower detection limits for all studies. This assumption lowered the estimates of exposure for the apple thinner and applicator children populations relative to the other populations. Comparison of the data below the 25th percentile is problematic because of these differences in detection limits.

### Data analysis.

In the 1995 study of applicator children, DMTP levels measured in the two samples (collected 3–7 days apart) were averaged to yield one value per child. This is essentially equivalent to the procedure employed in the 1999 farmworker and farmworker children study, where equal volumes of two or three samples (collected within 2 weeks) were pooled and the resultant composite sample was analyzed to yield one value per child. Two samples were also collected per child in the 1998 Seattle children study; the first in the spring and the second several months later (Lu et al. 2001) showed no difference in DAP metabolite levels related to season of sample collection, so these samples were averaged to yield one value per child. In a few cases for each of these studies, only one sample was provided or analyzed per child. In these instances, that sample was assumed to provide the best available estimate of the child’s exposure. It seems unlikely that these sampling differences could produce substantial differences for the analyses conducted.

Distributions of urinary DMTP concentrations (micrograms of metabolite per liter of urine) were created for the populations sampled in the three cross-sectional studies. These distributions were compared at various percentiles, and cumulative frequency distributions were created to describe the data. The metabolite levels for two of the populations were not normally distributed; therefore, nonparametric tests including the Wilcoxon matched-pairs signed-rank test for paired samples and the Mann-Whitney *U*-test for independent samples were used to determine significant differences between groups. All analyses were performed using the statistical package Stata 6.0 (StataCorp, College Station, TX) or SPSS 10.0.5 (SPSS Inc., Chicago, IL).

The three dimethyl DAP concentrations (dimethylphosphate, DMTP, and dimethyl-dithiophosphate) for each sample were converted to their molar equivalents and summed to produce a composite dimethyl alkylphosphate (DMAP) value (nanomoles of metabolite per liter of urine) for the Seattle children and the farmworker children to allow a more thorough comparison of these two populations. The Mann-Whitney *U*-test for independent samples was used to compare these groups.

Differences in sampling strategies between these three studies and the two repeated-measures studies precluded direct statistical comparisons across all groups. To assess the exposure of the apple thinner and farm community children study populations, we calculated the arithmetic mean DMTP level for each participant. Distributions of these mean concentrations were created and compared at various percentiles.

Creatinine was measured in most of these studies, but we chose not to adjust the values because of concerns regarding the validity of such adjustments, particularly for children (Barr et al. 2005).

## Results

**Figure 2:**
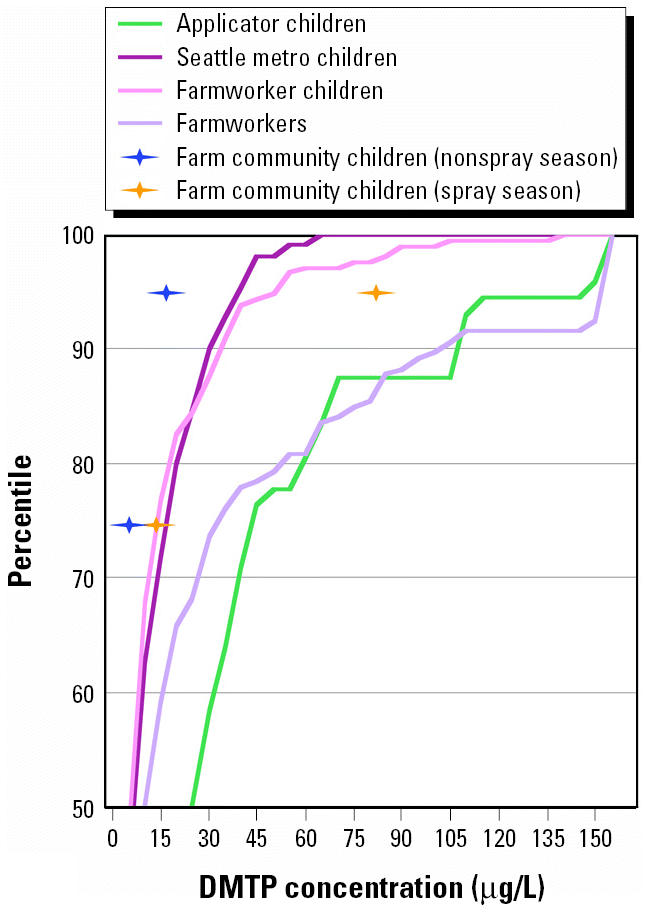
Cumulative frequency distribution of the top 50th percentile of urinary DMTP concentrations (μg/L) of children of pesticide applicators, children living in the Seattle metropolitan area, children of farmworkers, and adult farmworkers. Diamonds indicate the 75th and 95th percentiles for children living in farming communities during the nonspray season (blue) and the spray season (orange).

The five studies examined in this analysis included 437 children and 233 adults, who provided > 2,000 urine samples. Maximum values and selected percentiles for DMTP levels in the urine of the adult farmworkers, applicator children, farmworker children, and Seattle children in the cross-sectional studies are presented in Table 3. At the median (50th percentile), DMTP concentrations were highest in children of applicators (25 μg/L), followed by adult farmworkers (10 μg/L), and then children living in the Seattle area and children of farmworkers (6.1 and 5.8 μg/L, respectively). This pattern continued at the 75th percentile, with applicator children higher than adult farmworkers (44 vs. 32 μg/L), and Seattle children higher than farmworker children (17 vs. 13 μg/L). Even at the 90th percentile, the DMTP concentrations for applicator children were greater than for adult farmworkers (110 vs. 99 μg/L) and the other two groups. At the 95th percentile, however, this trend changed, with adult farmworkers having the highest concentrations (180 μg/L), followed by applicator children (130 μg/L), farmworker children (50 μg/L), and finally Seattle children (39 μg/L). The cumulative distributions for the top 50th percentile of these four populations are presented in Figure 2.

Metabolite concentrations in applicator children were higher than those in adult farmworkers (*p* = 0.02), although this difference was not statistically significant when correction for multiple comparisons was included in the analysis. DMTP concentrations in the urine of the applicator children were higher than those of either the farmworker children (Mann-Whitney *U*-test, *p* < 0.0001) or the Seattle children (Mann-Whitney *U*-test, *p* < 0.0001). Adult farmworker concentrations were higher than those of either the farmworker children (Wilcoxon matched pairs, *p* < 0.0001) or the Seattle children (Mann-Whitney *U*-test, *p* = 0.002). DMTP concentrations in farmworker children were not significantly different from those in Seattle children (*p* = 0.73). This information is summarized in Table 4.

We compared DMTP levels in these four populations qualitatively with DMTP levels in the NHANES III population survey (CDC 2003) for children 6–11 years of age (the youngest population sampled) and Mexican Americans (most of the adult farmworkers in the 1999 cross-sectional study were Hispanic). Table 3 presents DMTP levels for the 25th, 50th, 75th, 90th, and 95th percentiles. Applicator children had higher levels than did the NHANES III children at all percentiles. Farmworker children and Seattle children had higher DMTP concentrations at the 50th percentile than did the NHANES III children, but not at the higher percentiles. DMTP concentrations for the adult farmworkers were consistently higher than the NHANES III Mexican-American subgroup.

Table 5 presents the distributions for composite DMAP concentrations from the Seattle children and the farmworker children. DMAP concentrations were significantly higher for the Seattle children compared with the farmworker children (median values of 117 nmol/L and 87 nmol/L, respectively; *p* = 0.007). At the 50th percentile, DMAP concentrations were similar for the NHANES III children (6–11 years of age) and the farmworker children. At the 75th and 90th percentiles, the NHANES III values were similar to the Seattle children values and substantially higher than the farmworker children values. At the 95th percentile, the NHANES III values were substantially higher than either the Seattle children or farmworker children values.

Distributions of DMTP levels in the repeated-measures studies are presented in Table 6, which provides the 25th, 50th, 75th and 95th percentiles of the arithmetic mean urinary DMTP concentrations for the apple thinners, the farm community children (spray season), and the farm community children (nonspray season). DMTP concentrations among the apple thinners, who worked in fields soon after crop spraying, were one to two orders of magnitude greater than those of the farm community children. The 50th percentile DMTP concentration for the apple thinners (530 μg/L; Table 5) was > 50 times higher than that of the adult farmworkers (10 μg/L; Table 2). As reported by Koch et al. (2002), metabolite concentrations were significantly higher for the farm community children during the spray season than during the nonspray season.

Figure 2 includes data points describing the 75th and 95th percentiles of the arithmetic mean urinary DMTP concentrations for the farm community children during both the spray and nonspray seasons. Metabolite levels for the apple thinners were beyond the scale of this figure. Figure 2 demonstrates that, at the 75th percentile, all children except for the applicator children had roughly similar metabolite levels. At the 95th percentile, the farm community children (spray season) demonstrated higher levels than did the Seattle children, the farmworker children, and the farm community children (nonspray season). The maximum value for the farm community children (spray season) also exceeded the maximum value for the farmworker children [180 μg/L (Table 6) and 140 μg/L (Table 3), respectively].

## Discussion

The two most striking findings from this analysis were the relatively high levels of metabolites among applicator children compared with the other study groups and the similarity in metabolite levels between farmworker children sampled in 1999 and Seattle metropolitan area children sampled in 1998.

DMTP levels in children of pesticide applicators sampled in 1995 were significantly higher than those in the farmworker children in 1999, and were also higher than those in farmworkers in 1999 up to the 90th percentile. Direct comparison of these populations is problematic because pest control strategies may have changed over the 4 years that separated these studies. Pesticide use practices in this region are geared to many factors, including crop type, weather, pest infestation levels, and adoption of less chemical-intensive integrated pest management techniques. Additionally, the 1995 study sampled children during the active crop spraying season, whereas the 1999 sampling occurred for the most part after the peak spraying period.

**Figure 3:**
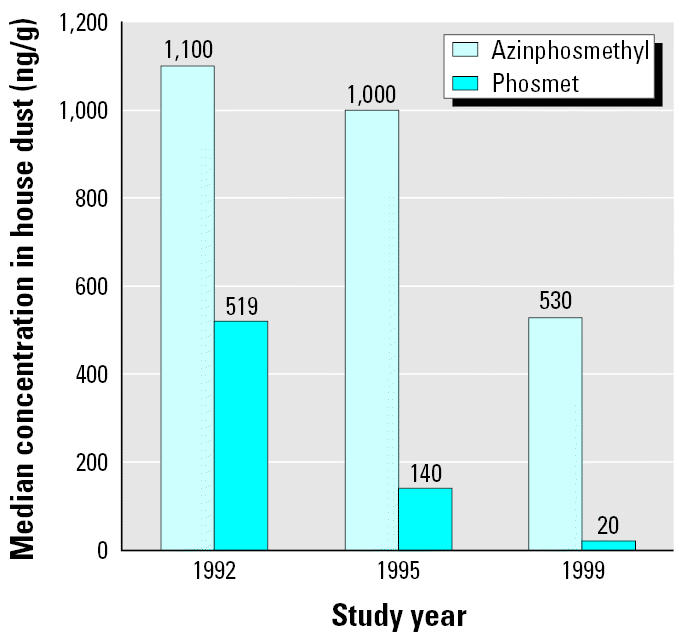
Median pesticide concentrations in house dust from households with agricultural workers in Washington State, 1992–1999. Data for 1992 from Simcox et al. (1995); for 1995 from Lu et al. (2000); for 1999 from Curl et al. (2002).

One way to gauge changes in pesticide use that is less affected by the time of sampling is to examine pesticide concentrations in household dust in these studies. Such an approach is based on the observation that pesticide concentrations in house dust are less susceptible to short-term fluctuations than are urinary metabolite measurements, and on the assumption that changes in pesticide levels in dust reflect changes in pesticide use practices and therefore differences in exposure opportunity. In a previous study, we noted that ethyl parathion concentrations in house dust decreased dramatically after this compound was withdrawn from agricultural use (Fenske et al. 2002). Figure 3 provides the median house dust concentrations of azinphos-methyl and phosmet—the primary dimethyl OP pesticides used in regional tree fruit production—from studies conducted in 1992 (Simcox et al. 1995), 1995 (Lu et al. 2000), and 1999 (Curl et al. 2002). These concentrations decreased over time: Azinphosmethyl concentrations in 1999 were about half those measured in 1995, and phosmet concentrations decreased even more dramatically. Other factors that might explain this difference include relatively high parental exposures during pesticide handling with consequent para-occupational exposure for the children, and the close proximity of the homes of many applicators to pesticide-treated farmland.

A second striking finding from this analysis was the lack of a significant difference between DMTP levels in farmworker children and Seattle children (Table 3) and the significantly higher DMAP concentrations among Seattle children compared with those in the farmworker children (Table 5). We had anticipated that the farmworker children would exhibit higher metabolite concentrations, given the widespread use of dimethyl OP pesticides in the Yakima Valley where they resided. The Seattle population consisted of 2- to 5-year-old healthy children living in the Seattle metropolitan area who had no known risk factors for pesticide exposure; less than half of the parents of these children reported use of any pesticides on lawns, gardens, indoors, or pets (Lu et al. 2001). The farmworker children were of a similar age, resided in the lower Yakima Valley about 150 miles east of Seattle, and were also considered a healthy population; all farmworker children lived with at least one adult actively engaged in farm labor (Thompson et al. 2003). It appears that most of these farmworker children had dimethyl OP pesticide metabolite concentrations lower than or indistinguishable from those of children living in urban and suburban environments whose parents did not work with pesticides. We have also examined the DMTP and DMAP metabolite concentrations of child subgroups in the Yakima Valley study based on the agricultural task of the adult farmworker (e.g., pesticide application, crop thinning) and did not find differences across these subgroups (Fenske et al. 2004). That the farmworker children did not have unusually high metabolite levels is further confirmed by comparison of these data with the 1999–2000 NHANES data for children 6–11 years of age (Tables 3, 5). Data comparisons across laboratories can be problematic, but in this case the CDC laboratory that analyzed the NHANES samples and the University of Washington laboratory that analyzed the present results had participated in a round-robin test for the DAP compounds, and these labs were found to produce comparable results (James et al. 2003).

DAP metabolite measurements probably reflect exposures that occurred in the previous 3–5 days. Most of the farmworker children’s samples were collected well after dimethyl OP pesticide crop spraying in the region, so the measured urinary metabolite concentrations did not necessarily capture peak exposures for this population that may have occurred in the late spring and early summer. The longitudinal study of children in an agricultural community reviewed here (Koch et al. 2002) provides persuasive evidence that DAP metabolite levels can increase during such spraying periods, and the 1995 study of children of pesticide applicators sampled during the active crop spraying period had higher metabolite levels than did other children in the same community (Loewenherz et al. 1997; Lu et al. 2000).

Our comparison of metabolite levels in farmworker children and Seattle children led us to conduct a dietary exposure study in the Seattle metropolitan area. We examined OP pesticide exposures in preschool children who consumed primarily organic juice and produce and those of children who consumed conventional (nonorganic) foods (Curl et al. 2003). We found that diet appeared to be the primary contributor to OP pesticide exposure among these children: The median DMAP concentration for children consuming organic juice and produce was about five times lower than for children with conventional diets (30 vs. 170 nmol/L, respectively). The median DMAP concentration for the farmworker children in the Yakima Valley study was 87 nmol/L. We speculate that a conventional diet rich with juices and fresh produce may have been more common among the Seattle children compared with the farmworker children. Supporting evidence for the importance of dietary differences was reported recently for a family in Germany (Heudorf et al. 2004). DAP concentrations were relatively low in a father and son compared with the mother’s levels. It was then learned that the mother had a special diet with a very high intake of fresh fruit. Substitution of supermarket fruit with organic (pesticide-free) fruit reduced the mother’s DAP concentration to those of her family members.

A third finding of this analysis was the 50-fold difference in DMTP concentrations between apple thinners and adult farmworkers. Although the apple thinner study was conducted in 1994, the metabolite levels measured at the time are considered representative of 1999 exposures, based on dislodgeable foliar residues measured at the time, allowable application rates, and U.S. Environmental Protection Agency estimates (Fenske et al. 2003). Biologic measurements of farmwork-ers’ pesticide exposure need to be collected during active work periods to capture peak exposures. It is interesting to note, however, that the adult farmworkers in the 1999 study had higher DMTP levels than did the NHANES III Mexican-American population, despite the fact that most farmworker samples were collected after peak exposures. This comparison supports the view that farmwork-ers represent an important subpopulation of Mexican Americans with respect to pesticide exposure.

Several other studies have employed DAP metabolites to estimate OP pesticide exposures among children. DAP concentration data from most of these studies have been compared in a recent publication (Barr et al. 2004a). A 1995 study in central Italy included collection of spot urine samples from 195 school children 6–7 years of age (Aprea et al. 2000). The primary findings of the study were that DAP concentrations were higher if pest control operations had been performed inside or outside the house in the preceding month, and that higher DAP concentrations were found for children than for a comparable adult population. Results were expressed as nanomole per gram of creatinine and so could not be compared directly with the present data. A 1998 study in Frankfurt am Main, Germany, involved collection of spot urine samples from residents in former U.S. Forces housing, including 309 children ≤5 years of age (Heudorf and Angerer 2001; Heudorf et al. 2004). These children had higher DAP concentrations than did adults (Heudorf and Angerer 2001). The median DMTP concentration among these children was 18.8 μg/L (Heudorf et al. 2004), exceeding the median concentrations of all of our study groups except applicator children. The authors concluded that the primary source of OP pesticide exposure in this population was from diet, and that these exposures in children may reach and even exceed the World Health Organization’s acceptable daily intake values (Heudorf et al. 2004).

A 1997 study of farmworker families near Fresno, California, included collection of spot urine samples from 9 children and 18 adults (Mills and Zahm 2001). Most samples did not have detectable levels of the six DAP metabolites. Frequency of detection was higher among children than among adults. The mean DMTP concentration (13 μg/L) for children was similar to the mean value (14 μg/L) found in the study of farmworker children in Washington (Curl et al. 2002).

A 2000 study in an agricultural community near the U.S.–Mexico border in southern Texas included collection of spot urine samples from 41 children (Shalat et al. 2003). Only eight of these samples (19.6%) had detectable levels of DMTP. The authors concluded that wipe samples of children’s hands may serve as a better exposure metric for epidemiologic studies than do house dust samples. Comparison with the Washington State studies was not possible because DMTP detection frequency was low and DMAP concentrations were not reported.

A 2000 study in Imperial County, California, focused on 20 children 11–17 months of age. The study examined the relationship between proximity of homes to treated farmland and DAP concentrations in the urine of the young children living in these homes (Royster et al. 2002). The median DMTP concentration reported was 3.2 μg/L, or about one-half that observed in our studies of farmworker and Seattle children. No significant difference was found between DAP concentrations of children living within 400 m (one-quarter mile) and those living more than 400 m from an agricultural field. It is not clear that the statistical analysis (nonparametric Mann-Whitney *U*-test) had sufficient power to detect a difference, given the sample sizes of five and nine, respectively. The authors did not compare DAP concentrations in the children living nearest to (< 400 m) and farthest from (> 800 m) farmland. It is interesting to note that the mean DAP concentration in the first group was 4.4 times higher than the mean concentration for the second group (123 vs. 28 μg/g creatinine), indicating that the most highly exposed children lived nearer to farmland. The 1995 study of applicator children in Washington State found that DAP concentrations were higher in children who lived closer to agricultural fields (Loewenherz et al. 1997; Lu et al. 2000), using several distance categories that were < 400 m, and a larger sample size. This study also focused on children more likely to spend time outdoors (2- to 5-year-olds) than did the Imperial County study (1- to 2-year-olds).

## Conclusions

This analysis makes evident that measurements of short-lived metabolites in urine show marked variability both within and across different studies. Children of pesticide applicators sampled during the active spraying season exhibited high DAP concentrations relative to other child populations. Children in an agricultural community without pesticide applicators in their households exhibited higher DAP concentrations during the active crop spraying season than during the rest of the year. Children of farmworkers sampled largely outside of the peak spraying season had DAP concentrations similar to or lower than those of children in the Seattle metropolitan area. This analysis highlights the importance of sample timing in biomarker studies of pesticide exposure and suggests that identification of high-exposure subpopulations in urban and rural communities can be challenging. Future studies should be designed as longitudinal investigations with frequent repeated measurements to capture peak exposures and characterize intrapersonal and interpersonal variability. This analysis also highlights the difficulty of designing epidemiologic studies to evaluate potential health effects of pesticide exposure in children. It cannot be assumed that children in agricultural communities have higher exposures than children in other environments, without taking into account crop spraying patterns and parental contact with pesticides. Studies that seek to categorize children’s exposure in these communities will need to sample both peak and nonpeak exposure periods and will need to evaluate multiple exposure pathways.

## Figures and Tables

**Table 1:** Characteristics of populations in five Washington State studies with cross-sectional sampling design.

	Location	Collection period	Sample size (*n*)	Samples collected	Age (years)	Relation to agricultural production	Sampling time frame
Applicator children	Wenatchee Valley	May–Jul 1995	72	137	2–6	Parent works as a pesticide applicator	During active spray season
Seattle metro children	Seattle metro area	May–Jun 1998 Oct 1998	110	207	2–5	None	NA
Farmworker children	Yakima Valley	Jul–Oct 1999	211	211^a^	2–6	Household member works as fieldworker of applicator	Most samples collected during nonspray season
Adult farmworkers	Yakima Valley	Jul–Oct 1999	213	213	≥19	Employed as a field worker or pesticide applicator	Most samples collected during nonspray season

NA, not applicable.

a Each of these samples represents a composite of equal volumes of two or three individual voids, each separated by a minimum of 3 days and all collected within a 2-week period.

**Table 2:** Characteristics of populations in five Washington State studies with repeated-measures sampling design.

	Location	Collection period	Sample size (*n*)	Samples collected	Age (years)	Relation to agricultural production	Sampling time frame
Apple thinners	Wenatchee Valley	May–Jul 1994	20	293	≥19	Employed as an apple thinner	During active spray season
Farm community children (spray season)	Wenatchee Valley	May–Jul 1998 Jun–Aug 1999	44	274	2–5	Reside in an agricultural community	During active spray season
Farm community children (nonspray season)	Wenatchee Valley	Dec 1997–Apr 1998 Aug 1998–May 1999	44	694	2–5	Reside in an agricultural community	During active spray season

**Table 3:** Urinary DMTP concentrations (μg/L) for participants from three cross-sectional Washington State studies and NHANES III data for children 6–11 years of age in the general U.S. population.

	Percentile						
Population	25th	50th	75th	90th	95th	97th	Maximum
Adult farmworkers	3.3	10	32	99	180	250	2,000
Applicator children	8.2	25	44	110	130	180	220
Farmworker children	2.4	5.8	13	33	50	57	140
Seattle children	2.4	6.1	17	29	39	42	60
NHANES^a^ children 6–11 years old	< LOD	4.1	20	40	62	—	—
NHANES^a^ Mexican Americans	< LOD	2.0	10	38	130	—	—

—, data not reported.

a Reported by CDC (2003).

**Table 4:** Statistical analysis of differences in urinary DMTP concentrations between populations.

	Population with higher exposure		
Comparison population	Adult farmworkers	Applicator children	Farmworker children
Applicator children	*p* = 0.02^a,b^	—	—
Farmworker children	*p* = 0.0001^c^	*p* < 0.0001^a^	—
Seattle children	*p* = 0.002^a^	*p* < 0.0001^a^	No significant difference^a^,^d^

a Mann-Whitney *U*-test for independent samples.

b Applicator children had higher DMTP levels than did adult farmworkers (*p* = 0.02), but this difference was not considered significant because of multiple comparisons (Bonferroni adjustment; *p* < 0.008 necessary for significance).

c Wilcoxon signed-rank test for paired samples.

d DMTP levels for Seattle children and farmworker children were not different (*p* = 0.73).

**Table 5:** Composite DMAP concentrations (nmol/L) for Seattle metropolitan area children, Yakima Valley farmworker children, and NHANES III children 6–11 years of age.

		Percentile				
Population	No.	25th	50th	75th	90th	95th
Seattle children	110	63	117^b^	250	453	545
Farmworker children	211	50	87^b^	174	378	522
NHANES^a^	471	23	91	270	460	679

a Data from Barr et al. (2004, Table 4).

b Significantly different, Mann-Whitney *U*-test, *p* = 0.007.

**Table 6:** Arithemetic mean urinary DMTP concentrations (μg/L) for participants in repeated-measures studies.

	Percentile^a^				
Population	25th	50th	75th	95th	Maximum
Apple thinners	310	530	610	1,100	1,100
Farm community children (spray season)	5.5	7.8	14	84	180
Farm community children (nonspray season)	3.8	5.5	9.5	18	45

a Percentiles of arithmetic means of repeated measures for individuals in study population.
